# Effects of Empagliflozin Combined with Anaerobic, Aerobic, and Endurance Swimming Protocols on Cardiac Structure and Electrophysiology in Healthy Rats

**DOI:** 10.3390/jcm15124773

**Published:** 2026-06-19

**Authors:** Samet Yavuz, Şahhan Kilic, Suha Asal, Mert Babaoglu, Cumaali Demirtaş, Mehmet Yildirim, Servet Altay, Ahmet Lütfullah Orhan

**Affiliations:** 1 Department of Cardiology, Ministry of Health Babaeski State Hospital, Kırklareli 39200, Türkiye; 2 Department of Cardiology, Ministry of Health Çorlu State Hospital, Tekirdağ 59850, Türkiye; drsahhankilic@gmail.com (Ş.K.); suhaasal@hotmail.com (S.A.); 3 Department of Cardiology, Sultan 2. Abdulhamid Han Training and Research Hospital, University of Health Sciences, Istanbul 34668, Türkiye; babaoglumert11@gmail.com (M.B.); lutfu.orhan@gmail.com (A.L.O.); 4 Hamidiye Experimental Animals Laboratory, Department of Physiology, University of Health Sciences, Istanbul 34668, Türkiye; cumaali.demirtas@sbu.edu.tr; 5 Department of Physiology, Hamidiye Faculty of Medicine, University of Health Sciences, Istanbul 34668, Türkiye; mehmet.yildirim@sbu.edu.tr; 6 Department of Cardiology, Faculty of Medicine, Trakya University, Edirne 22030, Türkiye; drservetaltay@gmail.com

**Keywords:** empagliflozin, SGLT2 inhibitors, exercise, echocardiography, electrocardiography, ventricular remodeling, animal experiment

## Abstract

**Objective**: Sodium–glucose cotransporter 2 (SGLT2) inhibitors, particularly empagliflozin, have attracted considerable attention because of their cardiovascular benefits beyond glycemic control. However, the interaction between empagliflozin and exercise-induced physiological cardiac remodeling in healthy individuals remains insufficiently understood. This study investigated the effects of different swimming exercise protocols (anaerobic, aerobic, and endurance), administered alone or in combination with empagliflozin, on cardiac structure and electrophysiology. **Methods**: Thirty-six male Sprague–Dawley rats were randomly assigned to six groups (*n* = 6 per group): anaerobic (An), aerobic (Ae), endurance (En), and the corresponding exercise groups combined with empagliflozin (An + Empa, Ae + Empa, and En + Empa). Empagliflozin was administered by oral gavage at a dose of 15 mg/kg/day for 30 days. Transthoracic echocardiography, electrocardiography (ECG), and gastrocnemius electromyography were performed at baseline and at the end of the study to assess cardiac remodeling, heart rate, and neuromuscular function. The study was carried out over a 30-day intervention period following ethics committee approval on 24 July 2024. **Results**: No significant between-group differences were observed in echocardiographic parameters before the intervention. On day 30, significant differences were identified among the groups in interventricular septal thickness at end-diastole (IVSd) (*p* = 0.027), left ventricular internal diameter at end-diastole (LVIDd) (*p* = 0.009), and end-diastolic volume (EDV) (*p* = 0.014). Bonferroni-corrected post hoc analysis showed that the aerobic exercise plus empagliflozin group differed from several exercise-only groups, particularly in parameters related to ventricular size and filling volume, including LVIDd and EDV (*p* < 0.008). On day 30, electrocardiographic repolarization-related parameters, including QT, QTc, JT, and Tpeak–Tend intervals, also differed significantly among the groups (all *p* < 0.05). In post hoc analysis, the anaerobic exercise group showed significant differences in QT and JT intervals compared with the aerobic and endurance groups (*p* < 0.008). In the anaerobic protocol, empagliflozin was associated with a reduction in heart rate compared with the corresponding control group (*p* = 0.019). No significant between-group differences were observed in EMG findings. **Conclusions**: Different exercise protocols induce distinct patterns of adaptation in cardiac structure and electrophysiology in healthy rats. Empagliflozin (15 mg/kg/day) may modulate exercise-induced cardiac responses in a modality-dependent manner; the most pronounced echocardiographic effects were observed in the aerobic protocol, whereas the effect on heart rate was observed in the anaerobic protocol. These findings highlight the need for longer-term and mechanistic studies to further clarify the effects of SGLT2 inhibitors on physiological cardiac remodeling.

## 1. Introduction

Regular physical activity is strongly associated with improved cardiovascular health and reduced all-cause mortality. Repetitive hemodynamic loading during exercise induces a series of structural and functional adaptations that enhance myocardial performance and efficiency, commonly referred to as the athlete’s heart [1,2,3,4,5,6]. This association is reaffirmed by recent international guidance, which confirms that regular physical activity reduces cardiovascular morbidity and all-cause mortality [7].

Physiological cardiac remodeling is a benign and adaptive process that differs fundamentally from pathological remodeling associated with hypertension or valvular disease, as it is not accompanied by cardiac dysfunction, fibrosis, or increased risk of sudden cardiac death [8,9]. The pattern of remodeling is largely determined by the predominant hemodynamic stimulus imposed by training. Endurance and aerobic exercise favor eccentric remodeling through sustained volume loading, resulting in proportional chamber enlargement and increased left ventricular end-diastolic diameter. In contrast, high-intensity anaerobic activity imposes a pressure load that promotes concentric remodeling with increased wall thickness and relatively preserved cavity size [10,11].

These modality-specific adaptations are not solely structural; they are also accompanied by distinct autonomic and electrophysiological changes. Endurance training commonly induces resting bradycardia via increased parasympathetic tone, whereas high-intensity exercise is associated with greater sympathetic activation and catecholamine-mediated effects on repolarization dynamics [12,13]. When a pharmacological agent modulates preload, afterload, autonomic balance, or myocardial ion handling, it may therefore alter both mechanical remodeling and electrical behavior in a modality-dependent manner.

Sodium–glucose cotransporter 2 inhibitors (SGLT2is), including empagliflozin, have emerged as widely used agents in the treatment of type 2 diabetes mellitus (T2DM), with strong evidence of cardiorenal benefit from large outcome trials [14]. Beyond glucose lowering, the proposed mechanisms underlying their cardiovascular benefits include reductions in preload through osmotic diuresis and natriuresis, improvements in myocardial energetics and substrate utilization, attenuation of oxidative stress and inflammation, and modulation of cardiomyocyte ion homeostasis, including Na+/H+ exchanger activity [15,16].

Although these mechanisms have primarily been studied in disease settings, such as T2DM, heart failure, and populations at high cardiovascular risk, the interaction between SGLT2 inhibition and physiological exercise-induced remodeling in the healthy state remains poorly defined. Specifically, it is unclear whether empagliflozin, when combined with anaerobic, aerobic, or endurance training, enhances, attenuates, or qualitatively modifies modality-specific cardiac adaptations and repolarization indices. Accordingly, the present study was designed to address this knowledge gap.

Empagliflozin was specifically selected for this investigation because, among the SGLT2 inhibitors, it carries the most robust cardiovascular-outcome evidence (EMPA-REG OUTCOME) and the best-characterized load- and ion-modulating mechanisms, including preload reduction through osmotic diuresis and inhibition of the cardiac Na+/H+ exchanger, that plausibly intersect with exercise-induced cardiac adaptation [14]. The decision to study a healthy heart was deliberate: although the established indications of SGLT2 inhibitors concern disease states, their use is increasingly extending to lower-risk and physically active populations, yet whether empagliflozin alters benign, physiological (exercise-induced) remodeling is unknown. Establishing whether the drug is neutral, additive, or modifying with respect to physiological adaptation in the healthy heart therefore provides a necessary reference point for interpreting its effects in disease and in active individuals, which constitutes the clinical significance of the present experiment.

The present study aimed to investigate the effects of different swimming exercise protocols (anaerobic, aerobic, and endurance), administered alone or in combination with empagliflozin, on cardiac structure and electrophysiology in healthy rats using a multimodal assessment strategy that included echocardiography, electrocardiography, and electromyography.

## 2. Materials and Methods

### 2.1. Ethical Approval

This study was approved by the Hamidiye Animal Experiments Local Ethics Committee (HADYEK) of the University of Health Sciences (approval number: 30145 dated 24 July 2024). All animals used in the experiment were obtained from the Hamidiye Animal Production and Research Laboratory of the University of Health Sciences, where the study was conducted. This study was reported in accordance with the ARRIVE (Animal Research: Reporting of In Vivo Experiments) guidelines (Table S1).

### 2.2. Study Design and Groups

The study was designed as a prospective, randomized, and assessor-blind experimental study. Following a 1-week acclimatization period in the laboratory, a total of 36 rats were randomized into 6 groups (*n* = 6), with 6 rats in each group.

Groups:

Group 1: Anaerobic exercise + SS (1 mL/day, 30 days);

Group 2: Aerobic exercise + SS (1 mL/day, 30 days);

Group 3: Endurance exercise + SS (1 mL/day, 30 days);

Group 4: Anaerobic exercise + Empagliflozin (15 mg/kg/day, 30 days);

Group 5: Aerobic exercise + Empagliflozin (15 mg/kg/day, 30 days);

Group 6: Endurance exercise + Empagliflozin (15 mg/kg/day, 30 days).

The sample size was determined a priori using the OpenEpi 3.03 online sample size calculator (www.openepi.com). The calculation was based on mean values and standard deviations derived from a reference study examining empagliflozin effects on skeletal muscle function in a rat model of heart failure with preserved ejection fraction [17]. Assuming 90% statistical power and a 95% confidence interval, the minimum required sample size was calculated as six animals per group. This number was considered both statistically adequate to detect meaningful between-group differences and ethically appropriate in accordance with the 3Rs principles of animal research. The study was approved by the ethics committee on this basis.

Throughout the 30-day experimental period, all groups received a once-daily oral gavage volume of 1 mL. The control groups (Groups 1, 2, and 3) received 1 mL of sterile saline (0.9% NaCl) daily, whereas the empagliflozin groups (Groups 4, 5, and 6) received empagliflozin at a dose of 15 mg/kg/day in a total gavage volume of 1 mL [18,19]. The design and protocol of the study are summarized in Table 1.

### 2.3. Animals and Housing Conditions

The study used 36 male Sprague–Dawley rats aged 16–20 weeks and weighing an average of 300 ± 20 g. The rats were housed in pathogen-free laboratory conditions in polycarbonate cages at 21 ± 3 °C, 65–70% humidity, and a 12 h light/12 h dark cycle; they were provided with standard feed and tap water. Cage maintenance was performed daily. Throughout the study, animals were treated humanely in accordance with the principles outlined in the “Guide for the Care and Use of Laboratory Animals.”

#### Exercise Protocols

**Figure 1:**
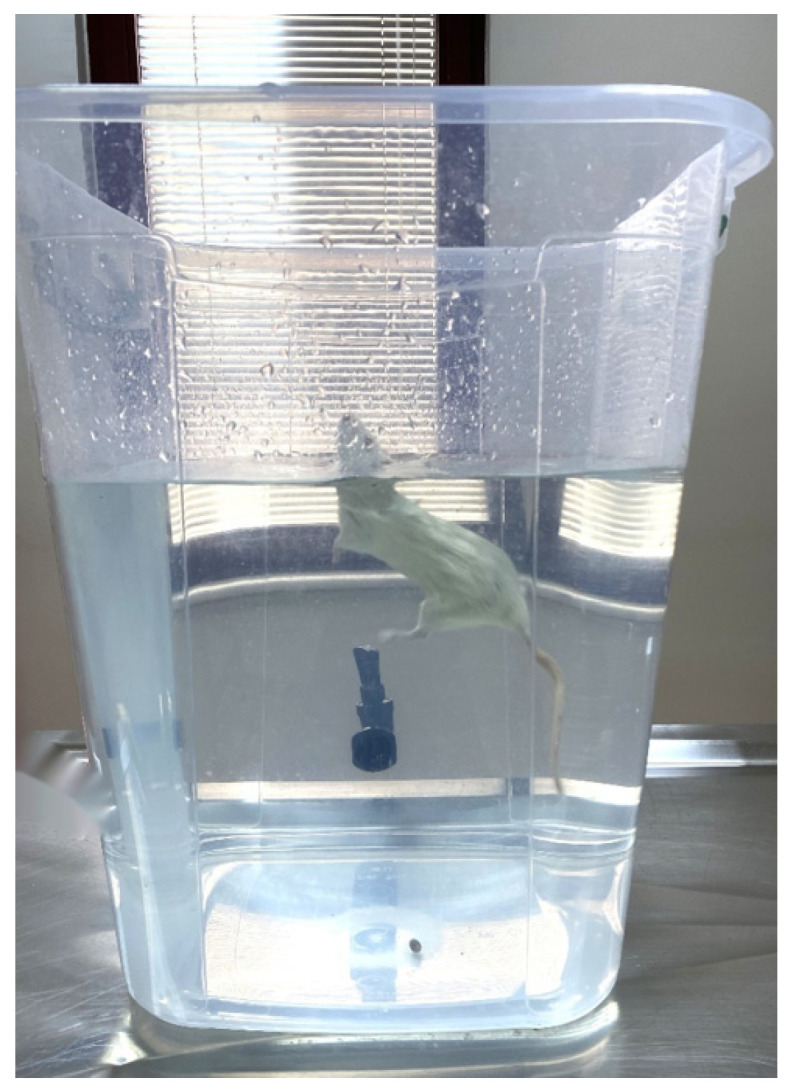
Swimming test in rats.

All exercise interventions were performed using a swimming test model in custom-built plexiglass tanks measuring 45 cm in height and 40 × 40 cm in width and length [20] (Figure 1). Water temperature was maintained at 23–26 °C, and the water was changed daily. To minimize procedure-related stress, the rats were acclimated to the aquatic environment and water temperature for 3 days before the initiation of the exercise protocols [21].

The anaerobic exercise training protocol consisted of ten 30 s sets, each followed by a 1 min rest period. This protocol was performed 4 times per week for 4 weeks, with a load equivalent to 50% of the rat’s body weight attached to the back. Because the load was too heavy for the rats to remain on the water surface, they were required to perform repeated jumps to breathe. During each 30 s set, each rat performed an average of 10 jumps [22,23].

In the aerobic exercise model, following a 1-week adaptation period, swimming duration was initiated at 10 min on the first day without any additional load and was increased by 10 min on each subsequent day until reaching 40 min/day. Thereafter, in the aerobic exercise protocol, the rats swam for 40 min/day, 5 days per week, for 4 weeks [24].

The endurance exercise protocol was designed as a high-intensity interval swimming model. In this protocol, rats swam for 20 min with a load equivalent to 15% of their body weight, followed by a 40 min rest period. This cycle was repeated 8 times [25].

### 2.4. Anesthesia

General anesthesia was administered prior to echocardiography, electrocardiography, and electromyography to protect animal welfare, reduce distress that may occur during the procedure, and ensure adequate immobilization. Induction was achieved with a combination of intraperitoneal ketamine HCl (80 mg/kg) and xylazine HCl (10 mg/kg) [26,27]. The depth of anesthesia was confirmed by monitoring for loss of the pedal withdrawal reflex throughout the procedure.

### 2.5. Transthoracic Echocardiography

**Figure 2:**
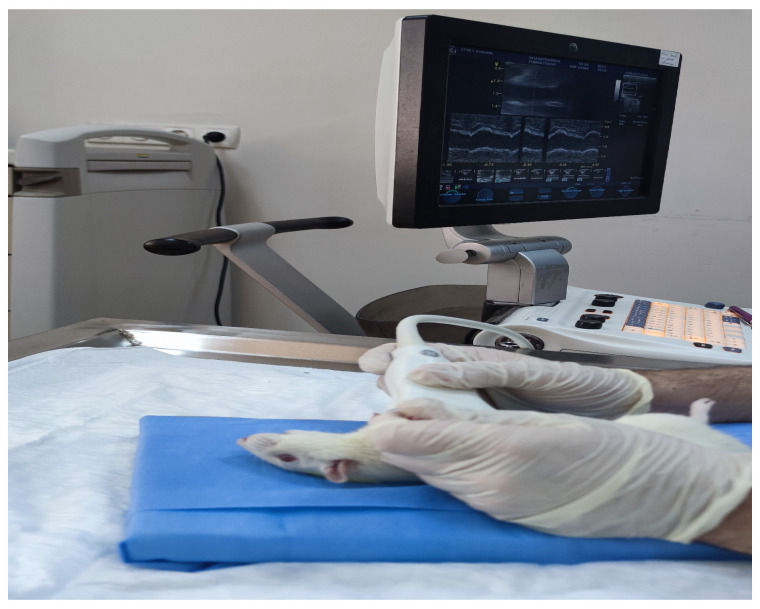
Echocardiographic assessment in rats.

Transthoracic echocardiography evaluation was planned on the 1st and 30th days of the experiment, i.e., before the first intervention and at the end of the study [27]. Following anesthesia induction, the chest area of each rat was carefully shaved to ensure optimal imaging. Two-dimensional, M-mode, and Doppler echocardiographic images were obtained using a General Electric Vivid S5 ultrasound system with a 12 L high-frequency linear transducer (GE Healthcare, Milwaukee, WI, USA) [28]. Rats were placed in the left lateral decubitus (side-lying) position. (Figure 2).

To calculate systolic function indicators, namely fractional shortening (FS) and ejection fraction (EF), standard parasternal long axis and short axis end-diastolic interventricular septum thickness (IVSd), end-systolic interventricular septum thickness (IVSs), end-diastolic left ventricular posterior wall thickness (LVPWd), end-systolic left ventricular posterior wall thickness (LVPWs), left ventricular internal diameter at end-diastole (LVIDd), left ventricular internal diameter at end-systole (LVIDs), ascending aorta systolic and diastolic diameters, aortic and pulmonary valve velocities, pulmonary valve velocity acceleration time (PAT), and aortic valve velocity deceleration time were measured. Diastolic function was assessed by measuring the ratio of early (E) to late (A) diastolic mitral flow velocities (Mitral E/A) [27,28,29,30].

### 2.6. Electrocardiography (ECG)

**Figure 3:**
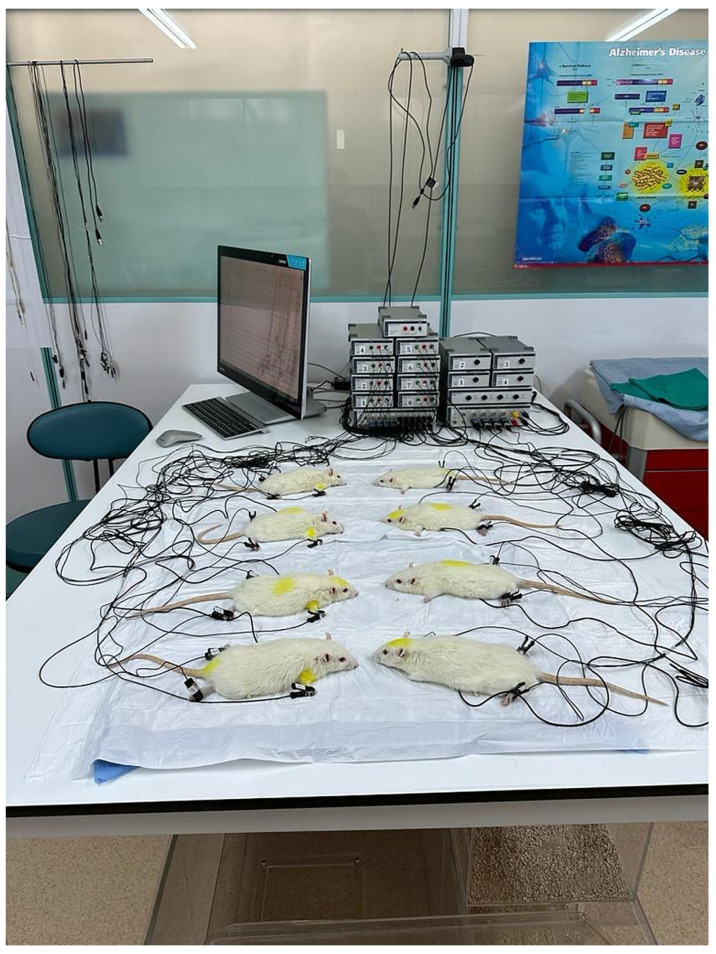
Electrocardiography (ECG) recording in rats.

At the beginning of the study and after the completion of the experiment, standard six-lead surface ECG recordings were obtained while the subjects were under general anesthesia (Figure 3). Alligator-type clamps(Local Supplier, Istanbul, Turkey) were placed on each of the four extremities to collect electrical signals. The PowerLab 16/35 system (AD Instruments, Castle Hill, Sydney, Australia) was used during the data acquisition process; recording parameters were fixed at 50 mm/s speed and 1 mV = 10 mm calibration values [27,31].

For electrocardiographic analysis, the following parameters were calculated from standard lead II recordings: heart rate (BPM), RR interval (ms), PR and QRS durations (ms), JT interval (ms), Tpeak Tend interval (ms), and QT interval (ms). Additionally, corrected QT (QTc) values were determined using the Bazett formula adapted to rat physiology [32].

### 2.7. Electromyography (EMG)

To analyze peripheral neuromuscular function, electromyography (EMG) recordings were obtained from the gastrocnemius muscle at both the beginning and end of the study [29,31]. While the subjects were under anesthesia, needle electrodes were placed in the body of the gastrocnemius muscle. The sciatic nerve was directly stimulated via a monopolar electrode to elicit the compound muscle action potential (CMAP) [33]. Recordings were obtained using the PowerLab 16/35 system with filter settings between 20 Hz and 2000 Hz. The following parameters were measured from the obtained waveform: onset latency (ms), peak latency (ms), and peak-to-peak amplitude (µV).

To minimize bias, the researcher who performed the ECG, ECHO, and EMG measurements and analyses was blinded to the group assignments of the animals.

**Figure 4:**
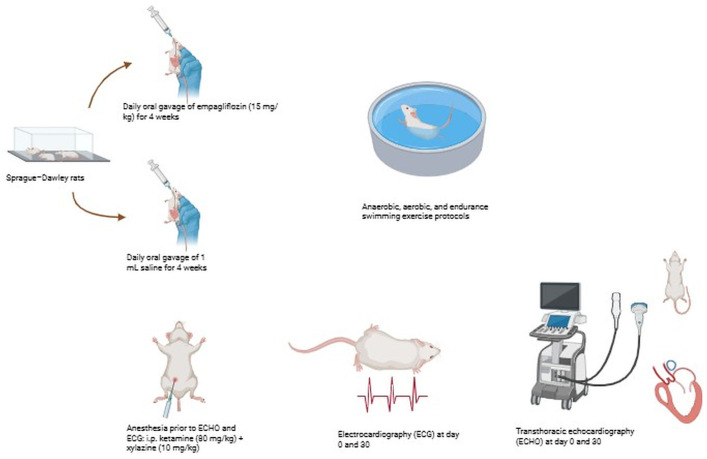
Schematic overview of the experimental design.

The experimental flow and procedures followed are exemplified in Figure 4.

### 2.8. Statistical Analysis

All statistical analyses were performed using SPSS version 27.0 (IBM Corp., Armonk, NY, USA) and Python (pingouin library, v0.5). Normality was assessed using the Shapiro–Wilk test. Because most variables violated normality assumptions at *n* = 6 per group, non-parametric methods were used as the primary analyses, with two-way ANOVA included as a supportive/exploratory analysis to evaluate interaction effects. Overall comparisons among the six experimental groups for each variable were performed using the Kruskal–Wallis test. When the Kruskal–Wallis test indicated a statistically significant difference (*p* < 0.05), post hoc pairwise comparisons were conducted using the Mann–Whitney U test. To account for multiple comparisons and reduce the risk of type I error, Bonferroni correction was applied; accordingly, a *p* value < 0.008 was considered statistically significant for pairwise comparisons. Comparisons between empagliflozin and control groups within the same exercise modality were performed as pre-planned exploratory analyses using the Mann–Whitney U test, supported by the significant exercise × drug interaction detected in the two-way ANOVA. Within-group comparisons were analyzed using the Wilcoxon signed-rank test. As a supportive exploratory analysis, two-way analysis of variance (ANOVA) was additionally applied to all primary outcome variables to explore the independent and interactive effects of exercise modality (anaerobic, aerobic, endurance) and drug treatment (control, empagliflozin) as between-subject factors. Post hoc comparisons within significant interactions were performed using Mann–Whitney U tests with Bonferroni correction (*p* < 0.017). Effect sizes were calculated using partial eta-squared (η^2^) for two-way ANOVA and rank-biserial correlation (r) for Mann–Whitney U tests, and 95% confidence intervals (CI) were reported alongside *p* values where applicable. Data were expressed as median (minimum–maximum) values. Unless otherwise specified, a two-sided *p* value < 0.05 was considered statistically significant.

## 3. Results

To confirm baseline comparability among the groups, transthoracic echocardiography was performed in all 36 rats before the start of the 30-day intervention period. The analyses showed no significant differences among the six groups in any of the cardiac variables examined. These findings indicate that randomization produced balanced groups and provided a common baseline for evaluating the effects of the interventions. Baseline measurements are presented in Table 2.

Echocardiographic analyses performed at the end of the 30-day intervention period demonstrated significant structural changes in the rat hearts associated with both exercise protocol and empagliflozin administration (Table 3). The Kruskal–Wallis test revealed an overall significant difference among the groups in left ventricular internal diameter at end-diastole (LVIDd) (*p* = 0.009) and end-diastolic volume (EDV) (*p* = 0.014).

**Figure 5:**
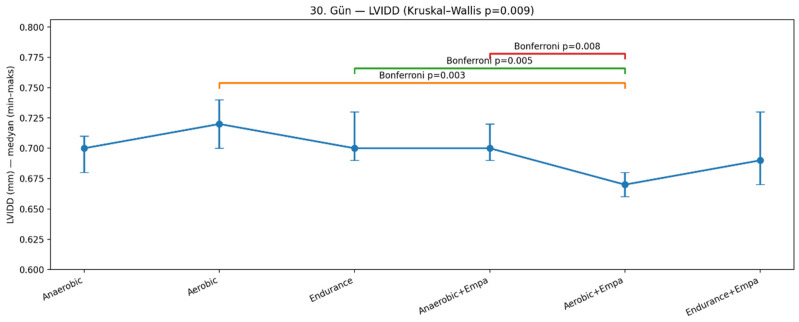
Left ventricular internal diameter at end-diastole (LVIDd) across groups at day 30.

Pairwise comparisons showed that the aerobic (Ae) group developed a significantly larger ventricular cavity than the anaerobic (An), anaerobic + empagliflozin (An + Empa), and endurance + empagliflozin (En + Empa) groups (*p* < 0.008) (Figure 5). This result may indicate that the aerobic training protocol, characterized by volume loading, is a strong stimulus for eccentric cardiac remodeling, consistent with previous reports. The greater ventricular dimensions observed in the aerobic training group, particularly when compared with some empagliflozin-treated groups, may indicate that empagliflozin modulates the remodeling response to aerobic exercise.

### 3.1. Electrocardiographic Findings

In our study, electrocardiographic parameters were compared among the groups at baseline, and no statistically significant differences were observed (Table 4).

**Figure 6:**
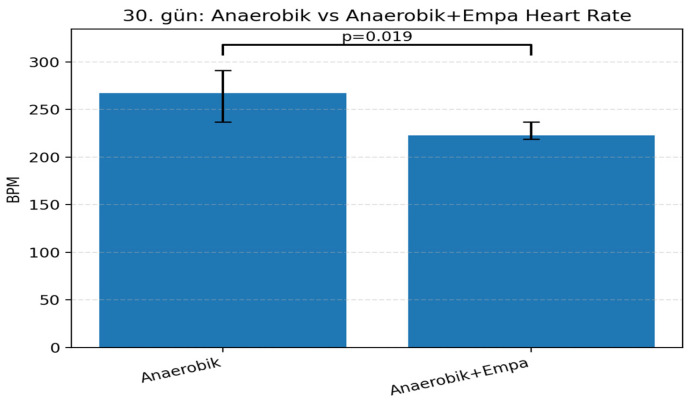
Two-Way ANOVA interaction plot showing exercise modality × empagliflozin effects on heart rate (anaerobic group only showed significant drug effect, *p* = 0.019) and QT interval (significant interaction, *p* = 0.004). An = Anaerobic, Ae = Aerobic, En = Endurance. Bars represent median ± IQR.

In the post-intervention electrocardiographic evaluation (Table 5), a statistically significant difference in heart rate was detected between the control and empagliflozin-treated groups within the anaerobic exercise model (*p* = 0.019) (Figure 6).

In contrast, within the aerobic and endurance exercise models, no statistically significant differences were observed between the control and empagliflozin-treated groups.

Overall, exercise appeared to induce modifications in the heart’s electrical conduction system and repolarization processes. Taken together, these findings suggest that exercise may influence cardiac electrical conduction and repolarization dynamics.

**Figure 7:**
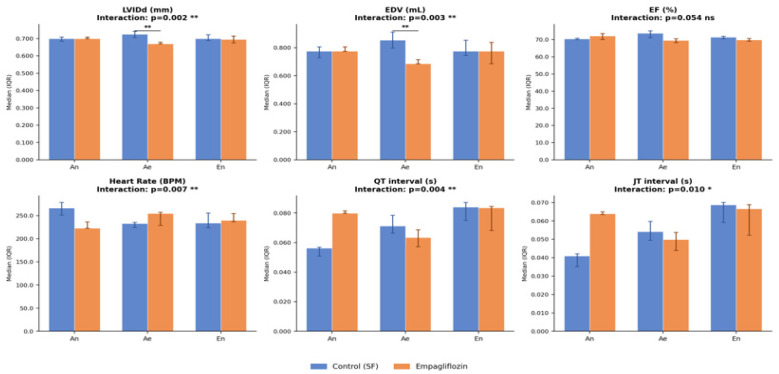
Two-way ANOVA interaction effects of exercise modality and empagliflozin treatment on selected cardiac outcomes at day 30. Upper panels: echocardiographic variables (LVIDd, EDV, EF). Lower panels: electrocardiographic variables (heart rate, QT interval, JT interval). Bars represent median values with interquartile range. Significance brackets indicate within-modality drug comparisons (Mann–Whitney U, Bonferroni corrected). Interaction *p*-values from two-way ANOVA are displayed in each panel title. An = Anaerobic, Ae = Aerobic, En = Endurance; SS = sterile saline (control). * *p* < 0.05, ** *p* < 0.01.

Two-way ANOVA revealed significant exercise × drug interaction effects for several primary outcome variables. In echocardiographic parameters, significant interactions were detected for LVIDd (F = 7.562, *p* = 0.002, η^2^ = 0.314) and EDV (F = 6.957, *p* = 0.003, η^2^ = 0.310), indicating that the effect of empagliflozin on ventricular dimensions and filling volumes was not uniform across exercise modalities but was specific to the aerobic protocol. Post hoc analysis confirmed that the drug effect within the aerobic group was significant for both LVIDd and EDV (*p* = 0.004), whereas no significant drug effect was observed within the anaerobic or endurance groups. In electrocardiographic parameters, significant interaction effects were found for heart rate (F = 5.886, *p* = 0.007, η^2^ = 0.282), QT interval (F = 6.754, *p* = 0.004, η^2^ = 0.310), QTc (F = 4.430, *p* = 0.021, η^2^ = 0.228), and JT interval (F = 5.343, *p* = 0.010, η^2^ = 0.263). Post hoc analysis showed that the drug effect on heart rate was significant only within the anaerobic group (*p* = 0.018), consistent with the non-parametric findings. These interaction effects confirm that empagliflozin does not exert a uniform cardiac effect but rather modulates exercise-induced adaptations in a modality-dependent manner. (Figure 7).

### 3.2. Electromyographic Findings

Electromyography (EMG) was conducted on the gastrocnemius muscle to evaluate the potential impact of the interventions on the peripheral neuromuscular system (Table 6). Statistical analysis revealed no significant differences among the six experimental groups across all measured EMG parameters (*p* > 0.05). Specifically, parameters including onset latency (time from stimulus to initial muscle response), peak latency (time to maximal response), and Compound Muscle Action Potential (CMAP) amplitude (total electrical magnitude of muscle fiber recruitment) remained consistent across cohorts [33]. These results suggest that neither the various exercise protocols nor empagliflozin administration induced detectable alterations in the electrophysiological properties of the skeletal muscle or its associated motor nerve over the 30-day intervention period [34,35]. The absence of observable neuromuscular alterations suggests that the main adaptations occurred at the cardiac level instead of the periphery.

## 4. Discussion

The present study investigated the synergistic effects of empagliflozin and distinct swimming exercise modalities, specifically anaerobic, aerobic, and endurance protocols on the cardiac architecture and function of healthy male Sprague–Dawley rats. By employing a multimodal diagnostic approach (ECG, ECHO, and EMG), we demonstrated that the interaction between empagliflozin and exercise does not elicit a uniform physiological response. Instead, the observed patterns of electrical and mechanical adaptation appear to be modulated by the specific metabolic and hemodynamic demands inherent to each exercise intensity.

The detection of differences between groups in repolarization-related parameters such as QT, QTc, JT, and Tpeak–Tend at day 30 suggests that exercise modality exerts a distinct effect on ventricular repolarization dynamics. In particular, the longer QT and JT intervals observed in the anaerobic group compared with the aerobic and endurance groups may reflect modality-specific differences in sympathetic activation and catecholamine-driven ion channel kinetics [36,37]. The biological significance of these differences, however, warrants careful interpretation. The absolute magnitudes of the observed QT and JT prolongations fall within ranges previously reported for physiological adaptation in healthy exercising rats, and no arrhythmic events were recorded. Repolarization intervals in rodents are substantially shorter than in humans and are highly sensitive to heart rate, autonomic tone, and body temperature; therefore, between-group differences in these parameters are more likely to reflect adaptive electrophysiological remodeling than to indicate a clinically meaningful proarrhythmic risk.

The reduction in heart rate (*p* = 0.019) in the anaerobic protocol with empagliflozin is noteworthy. These findings suggest that the clinical benefits of empagliflozin may extend beyond osmotic diuresis and preload reduction, potentially encompassing the modulation of sympathetic activity [38,39].

Empagliflozin has been shown to modulate cardiomyocyte ion homeostasis, including Na^+^/H^+^ exchanger activity, which may contribute to electrical stability under stress conditions [40,41]. Consistent with this, SGLT2 inhibitors have been proposed to reduce the risk of atrial arrhythmias in heart failure with preserved ejection fraction, potentially through similar electrophysiological mechanisms [42].

The attenuation of exercise-induced heart rate elevation observed in the anaerobic empagliflozin group may reflect the drug’s hemodynamic and ion-modulatory effects, although the precise mechanisms remain to be established in dedicated mechanistic studies.

In the echocardiographic evaluation, the emergence of intergroup differences in IVSd, LVIDd, and EDV parameters at the end of day 30 indicates that empagliflozin and exercise may interact through cardiac structural dimensions and filling volumes. In the post hoc analysis, the distinctiveness of the aerobic exercise + empagliflozin group from other groups, particularly in terms of LVIDd and EDV, can be explained by the addition of empagliflozin’s hemodynamic/metabolic contribution to the increase in cardiac efficiency developed with aerobic training.

The more pronounced reduction in end-diastolic diameter and volume in the aerobic empagliflozin group may reflect the additive effects of empagliflozin-induced preload reduction via osmotic diuresis and the favorable diastolic adaptations associated with aerobic training [43,44].

In the endurance protocol, the wall thickness changes observed with empagliflozin may be consistent with physiological hypertrophy, given that EF and FS remained preserved across groups [45,46]. Histological and metabolic validation will be required to confirm this interpretation.

Taken together, the findings suggest a protocol-specific interaction between the hemodynamic and metabolic demands of each exercise modality and the pleiotropic effects of empagliflozin. Notably, SGLT2 inhibitors have also been shown to favorably modify cardiac loading conditions in heart failure with preserved ejection fraction, further supporting their potential to modulate exercise-induced hemodynamic adaptations [47]. Whether these interactions involve oxidative stress, inflammation, NO bioavailability, or Ca^2+^ cycling pathways remains to be established in mechanistic studies [48,49,50].

In disease models, the cardiac effects of empagliflozin are well documented. In experimental heart failure with reduced ejection fraction, empagliflozin improves load-independent indices of contractility and diastolic function without necessarily altering fractional shortening, and it restores exercise endurance capacity in murine heart failure through enhanced skeletal-muscle fatty-acid oxidation [16,51]. This disease-model evidence contrasts with the present healthy-heart design, in which baseline systolic function (EF, FS) was preserved across all groups, indicating that the drug effect observed here reflects modulation of physiological adaptation rather than rescue of impaired function.

Evidence from diabetic and combined exercise–drug models further contextualizes these findings. A six-week endurance program combined with empagliflozin altered structural and functional cardiac indices in male diabetic rats [52], and combining swimming exercise with the SGLT2 inhibitor dapagliflozin produced the greatest cardioprotection in experimentally induced diabetic cardiomyopathy, with reductions in oxidative stress, inflammation, fibrosis and apoptosis and enhancement of autophagy [53]. At the level of physiological remodeling, both endurance and high-intensity interval training increase PI3K concentration and Hand2 gene expression and induce physiological hypertrophy [54]; this supports our interpretation that the modality-specific structural changes observed here reflect a benign, physiological hypertrophic program rather than pathological remodeling.

The clinical relevance of these observations should be framed cautiously. In the Empire Prevent Cardiac trial, 180 days of empagliflozin versus placebo did not change VO_2_ max, daily physical activity level, or quality of life in individuals with overweight or obesity at risk of heart failure [55]. Considered together with our results, this suggests that in the non-failing state, the functional impact of SGLT2 inhibition is limited, and that its principal value may lie in modulating—rather than augmenting—exercise-induced cardiac adaptation.

Exercise also acts through the bidirectional brain–heart axis. Structured training modulates central autonomic network activity and parasympathetic–sympathetic balance and increases neurotrophic signaling (e.g., brain-derived neurotrophic factor) that supports neurovascular coupling and cardiac regulation [56]. The autonomic component of this axis is consistent with the modality-dependent repolarization and heart-rate differences observed in our study and represents a promising target for future preventive strategies in cardiovascular disease.

Finally, empagliflozin is increasingly studied within combination cardiorenal strategies. In type 2 diabetic (db/db) mice, triple therapy combining an SGLT2 inhibitor with an endothelin-receptor antagonist on top of renin–angiotensin system blockade produced synergistic cardiac and renal protection, including reduced cardiomyocyte size and enhancement of the protective intrarenal ACE2/Ang(1–7)/Mas axis [57]. Although the present study did not include renal endpoints or such drug combinations, this broader context motivates future mechanistic work integrating exercise with combination pharmacotherapy.

## 5. Clinical Significance and Future Directions

From a translational perspective, these data indicate that exercise modality is an important determinant of how empagliflozin influences cardiac structure and electrophysiology, which is relevant for physically active and at-risk individuals who may receive SGLT2 inhibitors. The observed repolarization differences remained within ranges consistent with physiological adaptation, with no arrhythmic events recorded, and should not be interpreted as indicating proarrhythmic risk. Building on these data, future work should incorporate a sedentary control group, extend the follow-up period, and add histological, molecular and metabolic validation while extending the design to disease models to define the mechanisms underlying the modality-dependent drug–exercise interaction.

## 6. Limitations

Our study has several limitations. First, the absence of a sedentary control group limits causal interpretation of the observed adaptations, as it is not possible to fully disentangle the effects of exercise from baseline physiological variation. Second, the use of healthy male rats only limits the generalizability of the findings to clinical conditions such as diabetes, heart failure, or ischemic heart disease. Third, the 30-day follow-up may be insufficient to evaluate long-term cardiac remodeling processes. Fourth, the lack of histological, molecular, and metabolic validation makes it difficult to confirm the underlying mechanisms of the observed echocardiographic and electrocardiographic changes.

## 7. Conclusions

In conclusion, the combination of empagliflozin and exercise in healthy rats modifies cardiac electrical and mechanical adaptations depending on the protocol. In particular, the fact that empagliflozin produces a distinct pattern in filling volumes and ventricular dimensions in the aerobic protocol suggests that the drug’s hemodynamic and metabolic effects may work synergistically with exercise. Differences in repolarization markers on the ECG indicate that the type of exercise is an important determinant of electrical reorganization; empagliflozin may modulate this response via autonomic/ionic balance under certain conditions. This study contributes to a more nuanced understanding of the complex interaction between exercise physiology and cardiovascular pharmacology, opens new avenues for discovering strategies to optimize cardiac adaptation and function, contributes to the experimental literature on understanding the cardiac effects of SGLT2 inhibitors when used in conjunction with exercise, and establishes a foundation for more comprehensive translational studies.

## Figures and Tables

**Table 1:** Study design and group allocation.

Group	Exercise Modality	Daily Treatment (Oral Gavage, 1 mL, 30 Days)	*n*
1 (An)	Anaerobic	Sterile saline (SS, 0.9% NaCl)	6
2 (Ae)	Aerobic	Sterile saline (SS, 0.9% NaCl)	6
3 (En)	Endurance	Sterile saline (SS, 0.9% NaCl)	6
4 (An + Empa)	Anaerobic	Empagliflozin 15 mg/kg/day	6
5 (Ae + Empa)	Aerobic	Empagliflozin 15 mg/kg/day	6
6 (En + Empa)	Endurance	Empagliflozin 15 mg/kg/day	6

An, anaerobic; Ae, aerobic; En, endurance; Empa, empagliflozin; SS, sterile saline. All groups followed a prospective, randomized, assessor-blind design with a 1-week acclimatization period before the 30-day intervention.

**Table 2:** Baseline Echocardiographic Parameters.

Echo Variable	Anaerobic	Aerobic	Endurance	Anaerobic + Empa	Aerobic + Empa	Endurance + Empa	*p*-Value
IVSd (mm)	0.13 (0.125–0.14)	0.13 (0.12–0.135)	0.13 (0.12–0.13)	0.13 (0.12–0.135)	0.13 (0.125–0.13)	0.125 (0.12–0.14)	0.985
IVSs (mm)	0.17 (0.165–0.18)	0.16 (0.16–0.17)	0.165 (0.16–0.17)	0.17 (0.16–0.17)	0.16 (0.155–0.165)	0.16 (0.16–0.17)	0.259
LVIDd (mm)	0.72 (0.7–0.73)	0.70 (0.69–0.72)	0.71 (0.69–0.74)	0.71 (0.69–0.73)	0.70 (0.69–0.72)	0.72 (0.67–0.74)	0.984
LVIDs (mm)	0.465 (0.45–0.475)	0.455 (0.45–0.47)	0.465 (0.45–0.48)	0.46 (0.45–0.48)	0.46 (0.45–0.47)	0.47 (0.44–0.49)	0.770
LVPWd (mm)	0.12 (0.11–0.13)	0.12 (0.11–0.13)	0.12 (0.11–0.12)	0.12 (0.11–0.13)	0.12 (0.11–0.12)	0.12 (0.11–0.13)	0.976
LVPWs (mm)	0.25 (0.24–0.27)	0.25 (0.24–0.26)	0.25 (0.24–0.26)	0.25 (0.25–0.26)	0.25 (0.25–0.255)	0.25 (0.24–0.26)	0.900
AV Vmax (m/s)	1.27 (1.02–1.27)	1.12 (0.96–1.28)	1.25 (1.09–1.43)	1.1 (0.9–1.24)	1.23 (0.95–1.32)	1.19 (0.98–1.28)	0.561
Aortic acceleration time (ms)	22 (18–22)	21 (20–24)	19 (17–24)	22 (17–26)	24 (18–24)	26 (23–27)	0.499
AoD (mm)	0.29 (0.29–0.30)	0.29 (0.26–0.29)	0.28 (0.26–0.31)	0.29 (0.27–0.3)	0.27 (0.26–0.28)	0.29 (0.25–0.31)	0.299
AoS (mm)	0.34 (0.33–0.36)	0.33 (0.31–0.34)	0.32 (0.31–0.34)	0.32 (0.30–0.34)	0.31 (0.3–0.33)	0.31 (0.30–0.34)	0.313
PV Vmax (m/s)	0.79 (0.77–0.82)	0.81 (0.68–0.86)	0.75 (0.72–0.82)	0.79 (0.71–0.88)	0.77 (0.7–0.79)	0.78 (0.75–0.92)	0.761
Pulmonary acceleration time (ms)	31 (27–35)	33 (27–37)	32 (27–36)	26 (23–29)	26 (22–36)	25 (24–28)	0.206
Mitral E (m/s)	0.9 (0.76–0.99)	0.87 (0.82–1.04)	0.89 (0.83–1)	0.91 (0.79–1)	1 (0.9–1.06)	1.03 (0.95–1.15)	0.141
Mitral A (m/s)	0.32 (0.27–0.47)	0.36 (0.25–0.41)	0.33 (0.25–0.41)	0.32 (0.28–0.36)	0.37 (0.33–0.49)	0.29 (0.23–0.38)	0.603
LA diameter (mm)	0.44 (0.37–0.45)	0.40 (0.38–0.44)	0.43 (0.38–0.47)	0.43 (0.42–0.46)	0.39 (0.37–0.43)	0.44 (0.42–0.47)	0.386
EDV (mL)	0.837 (0.775–0.870)	0.774 (0.752–0.861)	0.79 (0.75–0.89)	0.82 (0.76–0.86)	0.77 (0.75–0.83)	0.82 (0.69–0.89)	0.982
ESV (mL)	0.246 (0.224–0.261)	0.231 (0.224–0.253)	0.25 (0.22–0.27)	0.24 (0.22–0.27)	0.24 (0.22–0.25)	0.25 (0.20–0.28)	0.998
FS (%)	34.9 (34.7–35.4)	35.2 (34–35.7)	35 (34.3–36)	35 (34–36)	34 (34–36)	34 (33–37)	0.943
EF (%)	70 (69–70.6)	70 (69–71)	70 (69–71)	70 (69–72)	69 (68–73)	69 (68–71)	0.926

Abbreviations: IVSd, interventricular septal thickness at end-diastole; IVSs, interventricular septal thickness at end-systole; LVIDd, left ventricular internal diameter at end-diastole; LVIDs, left ventricular internal diameter at end-systole; LVPWd, left ventricular posterior wall thickness at end-diastole; LVPWs, left ventricular posterior wall thickness at end-systole; AoS, aortic diameter at systole; AoD, aortic diameter at diastole; AV Vmax, peak aortic valve transvalvular velocity, PV Vmax, peak pulmonary valve transvalvular velocity; LA, left atrium;EF, ejection fraction; FS, fractional shortening; EDV, end-diastolic volume; ESV, end-systolic volume. Data are presented as median (minimum–maximum).

**Table 3:** Echocardiographic Parameters at Day 30 (Between-Group Comparison).

Echo Variable	Anaerobic	Aerobic	Endurance	Anaerobic + Empa	Aerobic + Empa	Endurance + Empa	*p*-Value	Subgroup Comparison *p* < 0.008	
IVSd (mm)	0.14 (0.135–0.145)	0.13 (0.13–0.135)	0.13 (0.13–0.135)	0.135 (0.13–0.137)	0.13 (0.125–0.13)	0.127 (0.124–0.14)	0.027	**-**	
IVSs (mm)	0.17 (0.17–0.18)	0.17 (0.17–0.18)	0.17 (0.17–0.18)	0.17 (0.16–0.17)	0.17 (0.16–0.17)	0.17 (0.16–0.18)	0.344	-	
LVIDd (mm)	0.70 (0.68–0.71)	0.72 (0.70–0.74)	0.70 (0.69–0.73)	0.70 (0.69–0.72)	0.67 (0.66–0.68)	0.69 (0.67–0.73)	**0.009**	**2 > 1** **2 > 6**	**2 > 4** **4 > 5**
LVIDs (mm)	0.45 (0.44–0.46)	0.46 (0.43–0.47)	0.455 (0.44–0.46)	0.45 (0.43–0.47)	0.44 (0.42–0.45)	0.45 (0.43–0.48)	0.770	-	
LVPWd (mm)	0.13 (0.12–0.13)	0.12 (0.11–0.13)	0.12 (0.11–0.12)	0.12 (0.12–0.13)	0.115 (0.11–0.12)	0.115 (0.11–0.13)	0.106	-	
LVPWs (mm)	0.27 (0.26–0.28)	0.26 (0.25–0.27)	0.27 (0.26–0.27)	0.27 (0.26–0.28)	0.26 (0.26–0.267)	0.26 (0.25–0.28)	0.440	-	
AV Vmax (m/s)	1.3 (1.23–1.37)	1.27 (1.15–1.51)	1.29 (1.1–1.43)	1.24 (1.14–1.34)	1.32 (1.18–1.39)	1.36 (1.25–1.54)	0.807	--	
Aortic acceleration time (ms)	26 (22–28)	25 (22–28)	21 (20–26)	24 (21–26)	20 (19–24)	25 (22–30)	0.236	-	
AoD (mm)	0.30 (0.27–0.30)	0.28 (0.26–0.29)	0.27 (0.27–0.28)	0.30 (0.26–0.31)	0.27 (0.27–0.30)	0.28 (0.27–0.31)	0.737	-	
AoS (mm)	0.32 (0.32–0.35)	0.33 (0.31–0.35)	0.32 (0.30–0.33)	0.33 (0.29–0.36)	0.3 (0.29–0.33)	0.31 (0.3–0.36)	0.683	-	
PV Vmax (m/s)	0.85 (0.79–0.95)	0.72 (0.63–0.76)	0.84 (0.71–0.92)	0.82 (0.79–0.94)	0.84 (0.78–0.89)	0.83 (0.75–0.89)	0.065	-	
Pulmonary acceleration time (ms)	29 (26–35)	31 (27–35)	30 (26–33)	35 (26–37)	33 (26–36)	31 (29–33)	0.818	-	
Mitral E (m/s)	0.98 (0.87–1.34)	1.01 (1–1.03)	0.93 (0.84–1.12)	0.90 (0.79–0.97)	0.89 (0.78–0.95)	0.8 (0.72–0.94)	0.077	-	
Mitral A (m/s)	0.35 (0.25–0.45)	0.37 (0.35–0.46)	0.37 (0.25–0.44)	0.27 (0.23–0.34)	0.26 (0.18–0.36)	0.26 (0.22–0.3)	0.068	-	
LA diameter (mm)	0.45 (0.40–0.48)	0.42 (0.41–0.46)	0.43 (0.38–0.44)	0.43 (0.39–0.48)	0.38 (0.38–0.46)	0.46 (0.4–0.48)	0.679	-	
EDV (mL)	0.774 (0.715–0.805)	0.854 (0.782–0.929)	0.77 (0.74–0.87)	0.77 (0.76–0.84)	0.69 (0.66–0.71)	0.77 (0.67–0.87)	**0.014**	**2 > 4**	
ESV (mL)	0.224 (0.210–0.238)	0.234 (0.197–0.251)	0.24 (0.21–0.24)	0.23 (0.19–0.25)	0.22 (0.18–0.22)	0.22 (0.19–0.26)	0.810	-	
FS (%)	35.2 (34.8–35.7)	38 (35.6–39.3)	36 (35–36.8)	37 (35–38)	34 (33–37)	35 (34–37)	0.159	-	
EF (%)	70.4 (69–71.1)	74 (71–75)	71 (70–72)	72 (70–74)	70 (68–73)	70 (69–72)	0.151	-	

Abbreviations: IVSd, interventricular septal thickness at end-diastole; IVSs, interventricular septal thickness at end-systole; LVIDd, left ventricular internal diameter at end-diastole; LVIDs, left ventricular internal diameter at end-systole; LVPWd, left ventricular posterior wall thickness at end-diastole; LVPWs, left ventricular posterior wall thickness at end-systole; AoS, aortic diameter at systole; AoD, aortic diameter at diastole; AV Vmax, peak aortic valve transvalvular velocity, PV Vmax, peak pulmonary valve transvalvular velocity; LA, Left Atrium; EF, ejection fraction; FS, fractional shortening; EDV, end-diastolic volume; ESV, end-systolic volume. Data are presented as median (minimum–maximum). Note: Bold values indicate statistically significant differences (*p* < 0.05).

**Table 4:** Baseline Electrocardiographic Parameters.

Ekg Variable	Anaerobic	Aerobic	Endurance	Anaerobic + Empa	Aerobic + Empa	Endurance + Empa	*p*-Value
RR interval (ms)	207 (188–242)	209 (173–217)	195 (181–253)	253 (207–276)	256 (226–275)	243 (209–327)	0.073
Heart rate (BPM)	303 (258–335)	307 (282–368)	335 (252–351)	303 (272–322)	269 (238–279)	279 (211–306)	0.139
PR interval (ms)	34 (32–35)	35 (31–36)	37 (32–38)	37 (36–38)	35 (33–37)	35 (32–37)	0.464
P duration (ms)	17 (14–17)	16 (15–16)	17 (14–21)	18 (17–19)	17 (16–18)	17 (15–18)	0.283
QRS interval (ms)	32 (31–35)	33 (32–37)	32 (32–34)	32 (32–34)	34 (32–35)	32 (31–34)	0.739
QT interval (ms)	49 (45–52)	49 (44–53)	47 (46–50)	46 (45–49)	52 (47–55)	48 (46–52)	0.516
QTc (ms)	106 (101–110)	107 (98–130)	109 (94–120)	100 (95–107)	101 (97–117)	96 (87–112)	0.562
JT interval (ms)	14 (12–15)	14 (11–20)	15 (13–16)	14 (12–15)	17 (15–18)	15 (13–18)	0.364
T peak T end interval (ms)	7 (6–8)	7 (5–12)	7 (6–8)	6 (4–6)	8 (7–9)	8 (6–9)	0.813

RR interval (ms): time between consecutive heartbeats; Heart rate (BPM): number of heartbeats per minute; PR interval (ms): atrioventricular conduction time; P duration (ms): duration of the P wave (atrial depolarization); QRS interval (ms): duration of ventricular depolarization; QT interval (ms): duration of ventricular depolarization and repolarization; QTc (ms): heart rate-corrected QT interval; JT interval (ms): repolarization duration obtained by subtracting the QRS interval from the QT interval; Tpeak–Tend interval (ms): time from the peak to the end of the T wave (index of repolarization homogeneity). Data are presented as median (minimum–maximum).

**Table 5:** Electrocardiographic Parameters at Day 30 (Between-Group Comparison).

Ekg Variable	Anaerobic	Aerobic	Endurance	Anaerobic + Empa	Aerobic + Empa	Endurance + Empa	*p*-Value	Subgroup Comparison *p* < 0.008
RR interval (ms)	225 (206–266)	258 (252–269)	257 (230–271)	269 (255–274)	235 (227–280)	250 (227–258)	0.181	
Heart rate (BPM)	267 (237–291)	233 (224–239)	234 (221–261)	223 (219–237)	255 (217–264)	240 (233–264)	0.055	**1 > 4**
PR interval (ms)	45 (42–50)	48 (46–53)	46 (46–50)	47 (46–49)	49 (46–55)	51 (43–54)	0.548	
P duration (ms)	17 (16–20)	18 (16–19)	16 (16–19)	17 (17–18)	17 (15–21)	20 (16–22)	0.790	
QRS interval (ms)	15 (14–18)	17 (16–18)	16 (15–16)	16 (15–18)	15 (13–16)	16 (15–16)	0.150	
QT interval (ms)	56 (50–57)	71 (63–80)	84 (71–89)	80 (65–85)	63 (54–74)	83 (61–87)	**0.010**	**1 > 2, 1 > 3**
QTc (ms)	110 (109–124)	140 (124–155)	170 (146–171)	157 (125–166)	134 (106–148)	165 (128–173)	**0.012**	
JT interval (ms)	41 (34–43)	54 (46–61)	69 (55–72)	64 (47–70)	50 (38–59)	67 (46–72)	**0.009**	
T peak T end interval (ms)	27 (22–30)	36 (28–44)	50 (36–53)	45 (29–52)	34 (25–41)	49 (28–55)	**0.045**	

RR interval (ms): time between consecutive heartbeats; Heart rate (BPM): number of heartbeats per minute; PR interval (ms): atrioventricular conduction time; P duration (ms): duration of the P wave (atrial depolarization); QRS interval (ms): duration of ventricular depolarization; QT interval (ms): duration of ventricular depolarization and repolarization; QTc (ms): heart rate-corrected QT interval; JT interval (ms): repolarization duration obtained by subtracting the QRS interval from the QT interval; Tpeak–Tend interval (ms): time from the peak to the end of the T wave (index of repolarization homogeneity). Data are presented as median (minimum–maximum). Note: Bold values indicate statistically significant differences (*p* < 0.05).

**Table 6:** (EMG). Data are presented as median (interquartile range). Empa, Empagliflozin. *p*-values derived from Kruskal–Wallis test.

Emg Variable	Anaerobic	Aerobic	Endurance	Anaerobic + Empa	Aerobic + Empa	Endurance + Empa	*p*-Value
Onset Latency (ms)	1.35 (0.7–1.5)	1.25 (0.9–1.5)	0.6 (0.6–0.7)	1.3 (0.35–1.3)	0.4 (0.25–0.85)	0.4 (0.35–2.15)	0.054
Peak Latency (ms)	2.7 (2.5–2.85)	2.4 (2.1–2.7)	1.3 (1.1–1.4)	2 (1.5–2.7)	2.1 (1.25–2.95)	2.5 (1.35–3.23)	0.053
Amplitude (mV)	10.01 (6.81–13.56)	14.19 (6.79–18.78)	15.03 (10.41–18.04)	7.44 (4.01–10.52)	8.39 (5.97–17.76)	7.42 (6.17–9.58)	0.073

## Data Availability

Data supporting the findings of this study can be obtained from the corresponding author upon reasonable request.

## Supplementary Materials

The following supporting information can be downloaded at https://www.mdpi.com/article/10.3390/jcm15124773/s1. Table S1: The ARRIVE guidelines author checklist.

## Abbreviations

3Rs	Replacement, Reduction, and Refinement (principles of humane animal research)
ACE2	Angiotensin-converting enzyme 2
Ae	Aerobic exercise group
Ae + Empa	Aerobic exercise plus empagliflozin group
An	Anaerobic exercise group
An + Empa	Anaerobic exercise plus empagliflozin group
Ang(1–7)	Angiotensin-(1–7)
ANOVA	Analysis of variance
AoD	Aortic diameter at diastole
AoS	Aortic diameter at systole
AV Vmax	Peak aortic valve transvalvular velocity
BPM	Beats per minute
CI	Confidence interval
CMAP	Compound muscle action potential
ECG	Electrocardiography (electrocardiogram)
ECHO	Echocardiography
EDV	End-diastolic volume
EF	Ejection fraction
EMG	Electromyography
Empa	Empagliflozin
En	Endurance exercise group
En + Empa	Endurance exercise plus empagliflozin group
ESV	End-systolic volume
FS	Fractional shortening
HADYEK	Animal Experiments Local Ethics Committee (Hayvan Deneyleri Yerel Etik Kurulu)
HFpEF	Heart failure with preserved ejection fraction
IVSd	Interventricular septal thickness at end-diastole
IVSs	Interventricular septal thickness at end-systole
JT	JT interval
LVIDd	Left ventricular internal diameter at end-diastole
LVIDs	Left ventricular internal diameter at end-systole
LVPWd	Left ventricular posterior wall thickness at end-diastole
LVPWs	Left ventricular posterior wall thickness at end-systole
Mitral E/A	Ratio of early (E) to late (A) diastolic mitral inflow velocities
Na+/H+	Sodium–hydrogen exchanger
PAT	Pulmonary valve velocity acceleration time
PI3K	Phosphoinositide 3-kinase
PR	PR interval (electrocardiography)
PV Vmax	Peak pulmonary valve transvalvular velocity
QRS	QRS duration (electrocardiography)
QT	QT interval
QTc	Corrected QT interval
RAS	Renin–angiotensin system
RR	RR interval (electrocardiography)
SGLT2	Sodium–glucose cotransporter 2
SGLT2i	Sodium–glucose cotransporter 2 inhibitor
SS	Sterile saline
T2DM	Type 2 diabetes mellitus
Tpeak–Tend	Tpeak-to-Tend interval
VO_2_ max	Maximal oxygen uptake
WHO	World Health Organization
η^2^	Eta-squared (ANOVA effect size)
